# Relationship between the Position of Impacted Third Molars and External Root Resorption of Adjacent Second Molars: A Retrospective CBCT Study

**DOI:** 10.3390/medicina55060305

**Published:** 2019-06-24

**Authors:** Dalia Smailienė, Giedrė Trakinienė, Augustė Beinorienė, Ugnė Tutlienė

**Affiliations:** 1 Department of Orthodontics, Lithuanian University of Health Sciences, LT- 50106 Kaunas, Lithuania; gyd_trakiniene@yahoo.com (G.T.); ugne.marmaite@gmail.com (U.T.); 2 Private Practice, LT- 50176 Kaunas, Lithuania; auguste.zorskaite@gmail.com

**Keywords:** cone-beam CT, impacted third molars, external root resorption

## Abstract

*Background and objectives:* Impacted third molars (ITM) are the most commonly-impacted teeth. There is a risk for ITM to cause a number of pathological conditions, and external root resorption (ERR) of adjacent teeth is one of the most prevalent. Retaining or prophylactic extraction of ITM is a polemic topic. External root resorption of adjacent teeth is one of possible indications for prophylactic removal of ITM. The aim of this study was to assess the relationship between external root resorption (ERR) on the distal aspect of second molars’ roots and positional parameters of ITM. *Methods:* Cone beam computed tomography scans of 109 patients (41 males, 68 females; mean age 26.4 ± 7.9 years) with 254 ITM (131 in the maxilla and 123 in the mandible) were retrospectively analyzed. Positional parameters of ITM (mesio-distal position, angulation, impaction depth, and available eruption space) were evaluated. The presence, location, and depth of ERR of adjacent second molars were assessed. *Results:* Analysis showed a relationship between ITM impaction depth, mesial inclination angle, and the presence of ERR. Mesial inclination angle of more than 13.6° increased the odds of ERR occurrence by 5.439 (95% CI, 2.97–9.98). ITM presence at the level of ½ of roots of the adjacent second molar or more apically increased the odds of ERR occurrence by 2.218 (95% CI, 1.215–4.048). No significant correlation was detected between the occurrence of ERR and patient age, gender, or the available eruption space in the mandible. Depth of ERR did not depend on its location. *Conclusions:* Incidence of ERR in second molars is significantly associated with mesial inclination and a deep position of ITM.

## 1. Introduction

Impacted third molars (ITM) are the most commonly-impacted teeth found in up to 24.4% of the population [1]. For orthodontists, ITM is also an issue of great importance. Partially-erupted third molars are evident in post-treatment records of 51% of orthodontic patients compared with 35% of untreated individuals [2]. Improvement of ITM angulation was observed in orthodontic patients treated with premolar extraction [3]. Conversely, distalization or uprighting of posterior teeth for creating space in the anterior segment leads to space deficiency in the posterior part of the arch [4]. 

Retaining or prophylactic extraction of ITM is a polemic topic among research groups [5]. Despite that, extraction of disease-free ITM remains one of the most common surgical procedures in oral surgery [6]. However, removal of ITM is associated with certain adverse effects. Short-term adverse effects include pain, swelling, trismus, temporary nerve damage, alveolar osteitis, infection, and secondary hemorrhage [5]. The most commonly-observed postoperative complications are pain, trismus, and swelling as a result of the local inflammatory process [6,7]. The peak of pain is observed during the time of 6–24 hours after the surgery [6,7]. A number of drugs have been studied for reduction of postoperative discomfort after third molar surgery by inhibiting the synthesis and/or release of the inflammatory mediators of acute inflammation. Non-steroidal anti-inflammatory drugs are the most widely used and have shown anti-inflammatory and analgesic effects [6]. However, in a recent study, Isola et al. found that treatment with a phytotherapeutic drug composed of herbal extracts also significantly decreased the incidence and severity of postoperative pain [7]. Long-term adverse effects of third molar surgery (permanent nerve damage (in up to 1% of cases) and damage to adjacent teeth during surgery) are less common [5].

Despite their bad position, ITM may be asymptomatic and cause no harm to the surrounding structures. However, there is a risk for them to cause a number of pathological conditions. Among various complications including pericoronitis, odontogenic cysts, tumors, periodontal diseases, and dental caries, external root resorption (ERR) is one of the most prevalent, even though it is quite difficult to diagnose [8,9,10,11,12,13,14]. 

Pressure-type external root resorption is a dental complication resulting from excessive osteoclastic activity provoked by excessive pressure of the impacted tooth or tumor on the adjacent tooth [15]. Unfortunately, pressure resorption is rarely clinically noticed before it progresses to the pulp area and causes specific endodontic symptoms such as pulpitis or periapical inflammation. If external root resorption is diagnosed too late, it can impede the prognosis of a tooth or even cause its loss [15]. Therefore, timely diagnosis of external root resorption is of great importance and should be performed as early as possible. 

Radiographically, the pressure resorption area can be identified adjacent to a stimulating factor such as an impacted tooth [15]. Three-dimensional imaging techniques have been demonstrated to be advantageous relative to two-dimensional radiography for ERR detection [16]. In the study by Oenning et al. [13], cone beam computed tomography (CBCT) images showed 4.3-times more ERR on second molars than did panoramic radiographs. Considering the fact that routine prophylactic CBCT scans are not ethical for prophylactic examination, it is important to detect the warning features of ITM position and select suitable patients for deeper examination with a CBCT scan for early diagnosis of pathology.

Previous studies have shown that ERR incidence on the second molar caused by ITM is related to patient gender, age, angulation, and depth of ITM [10,11,14]. Björk et al. [17] found that in 90% of mandibular ITM cases, retromolar space was inadequate. Concerning the results of previous studies showing that eruption space deficiency is related to third molar impaction, it might be hypothesized that space deficiency for ITM is related to the risk for it to cause ERR of the adjacent second molar.

Recent studies of ERR and ITM that were conducted using CBCT scans did not evaluate the relationship between ERR and ITM eruption space. Furthermore, in most previous studies, only mandibular molars were assessed [10,11].

Considering the wide variety of recommendations and opinions about the need for prophylactic extraction of ITM, as well as previous studies showing a relationship between ITM and ERR of adjacent second molars, it is important to determine the particular positional parameters of ITM that are most responsible for external resorption of the root of the adjacent second molar. This information could be valuable during orthodontic or surgical treatment planning. 

Therefore, the aim of the study was to assess the relationship between ERR of the distal surface of the root of the second molars and positional parameters of adjacently-impacted mandibular and maxillary third molars using CBCT scans. The null hypothesis of the study was that no relationship exists between particular positional parameters of ITM and incidence of ERR.

## 2. Materials and Methods

This study was a retrospective study. Before the study, ethical approval was obtained from the Regional Bioethics Committee (No. BE-2-12, 2015-02-09). The study was conducted in accordance with relevant guidelines and regulations. Written informed consent was obtained from each participant and from the parents/guardians of participants younger than 18 years old. 

All data were received from one private dental practice clinic database obtained from September 2014–August 2018 for diagnostic reasons (surgical removal of third molars, orthodontic assessment, etc.). The inclusion criteria were as follows: (1) the patients with no syndromes or other systemic diseases who had a CBCT scan with at least one ITM were included in the study sample; (2) considering the possibility that pressure ERR occurs in the presence of a contact of a second molar root with the crown of the impacted tooth [18], only patients with ITM having ITM with the mesial cusp tip at the cervical level of the second molar or more apically were included. 

The following exclusion criteria were specified: low quality of CBCT image, less than two thirds of ITM roots formed, patients with other pathologies (e.g., prosthetic crown, distal filling, root canal therapy of the second molar, tumor, cyst, etc.), second or first molars extracted or simultaneously impacted, materials of high density present in the region of second and third molars that could have influenced the accuracy of results.

Measurements were taken from CBCT scans which were performed according to the manufacturer recommendations using a medium-volume and high-resolution CBCT WhiteFox machine (de Götzen S.r.l., ACTEON Group, Olgiate Olona, Italy). The scanning parameters were set at 16 mA and 105 kV; a voxel size of 0.5 mm was fixed; and a field of view of 150 × 130 mm was set. The size of the slices performed in the CBCT was 0.2 mm. CBCT volumetric data were analyzed and reconstructed using the “WhiteFox” professional proprietary CBCT software. The analysis was performed during the period between August 2018 and September 2018.

All CBCT images were independently analyzed by examiners D.S. and U.T. The examiners navigated the CBCT volume to estimate the parameters of impacted molars and external root resorption of adjacent second molars. The examiners made the observations and estimations in three planes: sagittal, coronal, and axial. The unit of analysis was one tooth. The assessment of the mesial-distal position, impaction depth, and available eruption space in the lower arch was made in the sagittal section. The presence of ERR was evaluated in the axial plane and then confirmed in the sagittal and coronal planes. The depth of ERR was measured in the sagittal and axial planes, and then, the middle value was recorded.

The interobserver agreement was assessed by evaluating the results of the analysis of the two examiners. The level of agreement between the 2 observers was excellent (*k* = 0.855; *p* < 0.05). Doubts regarding the presence of ERR were resolved by consensus, and when a consensus could not be reached, the sample was excluded from the study. 

Positional parameters of ITM, including mesio-distal position, impaction depth, and available eruption space in the lower arch were evaluated. In addition, the presence, location, and depth of ERR of adjacent second molars were assessed. 

The mesio-distal position of ITM was categorized according to the method of Ventä et al. [19]. The angulations were classified as follows: vertical (±10° to the long axis of the second molar), mesioangular (+11–70° to the long axis of the second molar), distoangular (−11–70° to the long axis of the second molar), horizontal (≥71° to the long axis of the second molar). 

Depth of impaction was classified as:

- Sector A: the mesial cusp tip of ITM is at the cervical margin of the adjacent second molar.
- Sector B: the mesial cusp tip of ITM is at the level of ½ of roots of the adjacent second molar or more apically.

The available eruption space in the mandible was measured as the mesio- distal distance between the distal surface of the adjacent second molar and the ascending anterior border of the mandibular ramus. Space was categorized as:

- sufficient (space ≥ mesio-distal width of ITM)
- reduced (space > ½, but less than the entire mesio-distal width of ITM)
- insufficient (space < ½ of the mesio-distal width of ITM)
- no space (ITM is fully covered by the bone of the mandibular ramus)

**Figure 1:**
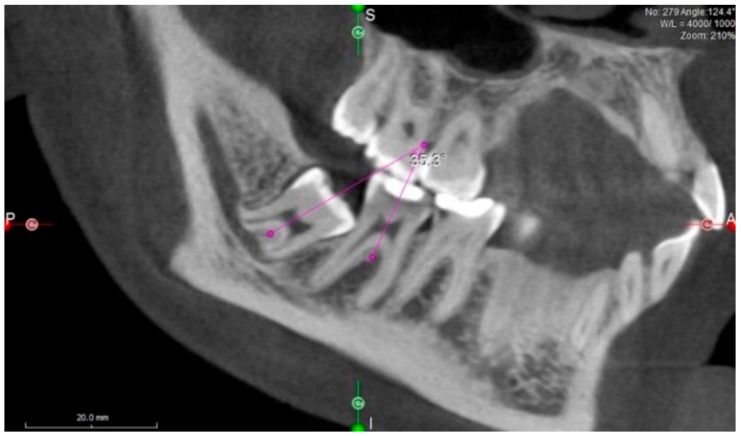
Measurement of impacted third molar angulation. Mesial angulation of impacted third molars (ITM) to second molars was assessed by drawing the axial lines of these teeth through their radiological center of the occlusal surface and furcation and measuring the angle between them.

Mesial angulation of ITM with adjacent second molars was assessed by drawing the axial lines of these teeth through their radiological center of occlusal surface and furcation and measuring the mesiodistal tipping angle between them (irrespective of the tooth buccolingual position) (Figure 1).

**Figure 2:**
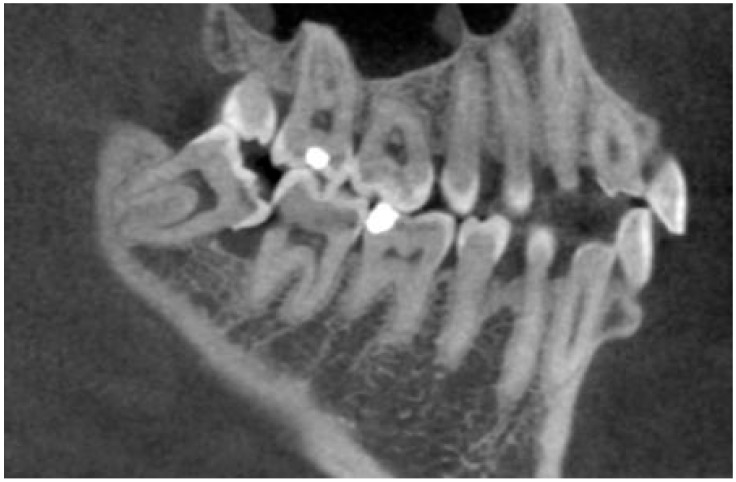
External root resorption of the lower second molar.

The presence of ERR was evaluated by navigating the volume of CBCT and exploring all sections of the second molar in all dimensions and was recognized as an apparent loss of substance on the distal surface of the adjacent second molar root. Suspicion of resorption was registered if a contact was seen between the third and second molars in addition to a change in the shape of the surface of the second molar (Figure 2). External root resorption of adjacent second molars was differentiated from carious lesions based on their radiographic appearance: in all cases where the change in the shape of the surface of the second molar was observed, but there was a clear gap between second molars and the dental crown of the third molar, this lesion was diagnosed as caries, and the tooth was recorded as free of ERR. 

Based on location, ERR was categorized into cervical (ERR present between the cervical part and 1/3 of the adjacent second molar root), medial (ERR present at the area between 1/3 and 2/3 of the adjacent second molar root), and apical (ERR present at the area between 2/3 to the apex of the adjacent second molar root). 

The severity of ERR was classified according to Ericson and Kurol [18]:

- slight: up to half of the dentine thickness is involved
- moderate: resorption midway to the pulp or more, the pulp lining being unbroken
- severe: the pulp is exposed by resorption.

The depth of ERR was measured as the longest distance between the deepest part of the defect and the point on the root surface that had a direct contact with ITM. 

### 2.1. Sample Size Estimation

The sample size calculation was based on the incidence of ERR of second molars caused by ITM [11,13]. The minimum expected incidence of ERR was 20.17%. To reach the power of 90% with an alpha of 5%, a minimum of 240 teeth were required. 

Initial analysis of 930 CBCT scans was carried out. According to the inclusion criteria, CBCT data of 109 patients with 254 ITM (131 (51.6%) in the maxilla and 123 (48.4%) in the mandible) were retrieved. CBCT data of 41 males (37.6%) and 68 females (62.4%), mean age 26.4 years (SD = 7.9), median of 23 (21–31) range 16–66 years, were analyzed.

### 2.2. Statistical Methods

The normality of the data distribution was tested with the Kolmogorov–Smirnov test. A non-parametric Mann–Whitney U-test was used for comparison of data in case of non-normality. Hypotheses of interrelations between characteristics were verified using the chi-squared test, Kruskal–Wallis test, and Spearman correlation coefficient. The interobserver agreement of CBCT findings was estimated by Cohen’s kappa coefficient and paired *t*-test. Logistic regression analysis and receiver operating characteristic (ROC) curve analysis were done to evaluate prognostic values for the prevalence of ERR in second molars. The value of *p* < 0.05 was considered statistically significant. Statistical analyses were made using SPSS Version 23 for Windows.

## 3. Results

A contact between the crown of ITM and an adjacent second molar was observed in 230 (90.6%) cases with significantly greater prevalence in the upper dental arch (126 (96.2%) ITM in the maxilla and 104 (84.6%) ITM in the mandible, *p* = 0.002).

The presence of ERR on the distal aspect of the second molar was identified in 102 teeth presented in 76 CBCT images, with overall prevalence of 40.2% (ERR detected in 44 (33.6%) upper second molars and 58 (47.2%) lower molars; *p* = 0.028).

There was no statistically-significant influence of patient age on the incidence of ERR (*p* = 0.732). The sample was divided into age groups based on age median: 23 years (group <23 years, *n* = 137, mean age 20.96 years (SD = 1.70); and group >23 years, *n* = 130, mean age 32.03 years (SD = 7.58)). However, the chi-squared test showed no relationship between patient age and ERR incidence (*p* = 0.837). 

The results of the evaluation of risk factors for the incidence of ERR are summarized in Table 1. There was a statistically-significant influence of third molar medial inclination angle, position, and lower molar impaction depth on the incidence of ERR. 

The ROC curve analysis disclosed that the cut-off value of mesial inclination angle was 13.6° (sensitivity 58.3, specificity 79.6, area under the curve 73.1%, *p* < 0.001). ERR was diagnosed in 58.3% of second molars when ITM were inclined more than 13.6° and only in 20.4% of cases when ITM were inclined less than 13.6°. Logistic regression analysis revealed that a mesial inclination angle of more than 13.6° increased the odds of ERR occurrence by 5.439 (95% CI, 2.97–9.98). 

Analysis also showed a relationship between lower third molar impaction depth and presence of ERR (*p* < 0.009, Table 1). Univariate logistic regression analysis revealed that ITM presence in Sector B increased the odds of ERR occurrence by 2.218 (95% CI, 1.215–4.048). Multivariate logistic regression analysis was not performed, because Spearman correlation analysis showed multicollinearity between variables (impaction depth and medial inclination angle, *r* = 0.5, *p* < 0.001).

No significant correlation was detected between the occurrence of ERR and patient gender (*p* > 0.05) or the available eruption space in the mandible (*p* > 0.05).

The location and severity of ERR of second molars are shown in Table 2. Of all 102 molars with ERR, 76 (74.5%) were identified with slight resorption, 26 (19.6%) with moderate, and 6 (5.9%) with severe resorption. Concerning location of resorption, 48 (47.1%) ERR cases were detected in the cervical, 40 (39.2%) in the medial, and 14 (13.7%) in the apical region of second molars. 

There was a significant association between ERR severity and third molar impaction depth (*p* < 0.001) (Table 3). 

Concerning ERR location and ERR severity, there was a significant correlation found (Spearman correlation coefficient *r* = 0.439, *p* < 0.001, Table 4). 

The mean depth of ERR was 0.67 (0.26) mm (ranging from 0.3–2 mm). However, no significant correlation between ERR location and ERR depth, measured in millimeters, was found (*p* = 0.27). 

## 4. Discussion

The results of the recent study showed that in the case of third molar impaction, in more than 90% of cases, there was a contact between the crown of ITM and the adjacent second molar, and consequently, this may result in ERR of the second molar.

Previous studies that used CBCT revealed the incidence of ERR on the second molar adjacent to ITM ranging from 20.17%–9.43% [10,11,12]. In the recent study, the presence of ERR was identified in 40.2% of cases, which was generally in line with previous findings. The explanation for the high prevalence of ERR in the present study may be that the study sample included many deeply-impacted ITM posing the highest risk of ERR. It is important to note that this high incidence of resorption found may not represent the incidence of ERR in an average population because the selection of study material included more severe than average cases.

Results of the present study showed that incidence of resorption was almost 15% higher in the mandible than in the maxilla. Oenning et al. [13] showed similar findings with lower ERR prevalence in the maxilla (14.3%) than in the mandible (31%). The main cause of third molar impaction in both jaws is inadequate space. However, due to prolonged growth of maxillary tuberosity and greater mesialization of maxillary dentition, there is more space for buccal eruption of upper third molars [20]. These findings explain why retention in the maxilla is less commonly observed than in the mandible. 

Furthermore, the recent study evaluated the ITM-related risk factors of ERR. 

One of the ERR risk factors discussed in the literature is patient age. Eruptive tooth movement does not stop after root formation is completed, and these teeth continue to exert mechanical pressure on adjacent second molars, thereby creating conditions for ERR progression [21]. Therefore, it can be assumed that the odds for ERR may increase with patient age. Previous research data indicated that patients older than 24 years have a relatively higher ERR prevalence and severity [10,11,12,22]. Wang et al. [11] found that an age of more than 35 years was an independent risk factor for ERR. However, the data of this study did not support the association between patient age and ERR. Ericson and Kurol [18] in their study of ERR of incisors caused by impacted canines also found no correlation between resorption and patient age. Severe resorption exposing the pulp was observed even in early stages of canine eruption.

The present study revealed that mesioangular and horizontal positions of third molars had the strongest association with ERR (50% and 84.2%, respectively). However, ITM in vertical and distoangular positions also tended to cause ERR (23.6% and 6.9%, respectively). According to the literature, mesial and horizontal inclination of ITM was most frequently related to ERR of second molars [13]. Matzen et al. [23] found that the vast majority of resorption cases were seen in relation to mesioangulated (71%) or horizontally-positioned (26%) third molars. Wang et al. [11] supposed that mesial and horizontal inclinations of ITM have a relatively large contact area between the second and third molars, which creates conditions for more pressure resorption. Oenning et al. [24] using finite element analysis established that ITM in close proximity with the adjacent tooth can generate areas of compression concentrated at the site of contact, and the major stress and amount of deformation occurred in the second and third molar regions in the situation of the horizontal position of ITM. In the present study, only the angle of ITM in the mesiodistal direction was measured, not taking into account the buccolingual inclination of the same tooth because only tipping of ITM in the sagittal direction can potentially cause a contact with the adjacent second molar, which was situated mesially to the third molar.

In the present study, a statistically significant influence of mesially-inclined ITM angle on the incidence of ERR was found. Nemcovsky et al. [14] concluded that in cases of non-erupted teeth, mesial inclination of 60° or more was significantly associated with ERR. However, the cut-off value of mesial inclination angle found in the present study was only 13.6°.

The other factor for ERR occurrence could be the available eruption space for ITM. The most common possible cause for mandibular third molar impaction is lack of space in the retromolar area [17]. One of the aims of this study was to evaluate if retromolar space deficiency also could be related to a higher possibility of adjacent teeth ERR. However, no significant correlation was detected between the occurrence of ERR and available space in the mandible. It is important to emphasize that in only 5.6% of cases, eruption space was sufficient, in 53.2% reduced, and in 31.55% insufficient, and in 9.7%, there was no space for the tooth to erupt. The available eruption space in the maxilla was not investigated in the present study because upper third molars are less limited by anatomic structures and there is more space for their eruption [20]. Jung and Cho [25] found that for 49% of maxillary molars, the eruption space was sufficient, and for 37%, it was reduced.

The study results showed a significant relationship between depth of mandibular ITM and occurrence of ERR. Even 66.7% of third molars having their mesial cusp were at the level of 1/2 second molar roots or more apically (Sector B), inducing ERR.

Summarizing the findings concerning ITM-related risk factors of ERR, the most important predictor of possible ERR was mesial inclination of ITM. Depth of impaction was an important predicting factor only in the lower dental arch, and space deficiency was not related to ERR.

The most common locations of ERR were the cervical and medial part of second molar roots. In the maxilla, most ERR defects were localized in the medial part of the root (54.5%), while in the mandible, most ERR defects were positioned in the cervical part (62.1%) (*p* = 0.002). Findings by Wang et al. [11] and Katkar [12] suggested that cervical area and root apical region might be susceptible regions for ERR. Contrarily, Nemcovsky et al. [14] stated that the apical region is the most susceptible region for ERR in individuals with completely unerupted third molars. 

Concerning the relationship between the location of ERR and its severity, the present study showed that slight ERR was usually found in the cervical area, while most cases of severe ERR were mostly detected in the apical part of the affected root. However, no significant correlation was found between ERR location and quantitative ERR depth (measured in millimeters) (*p* = 0.27). This means that the depth of ERR did not depend on its location. This mismatch between findings can be explained by the fact that the closer we get to the cervical part of the root, the thicker is the layer of the dentine. Therefore, the same quantitative depth of ERR (e.g., 1 mm) may be referred to as slight in the cervical part of the root, while it might be referred to as severe in the apical part of the root due to the thin layer of dentine in this location. However, the authors stand for the opinion that in the clinical aspect, it is more important to draw attention to the depth of ERR, which was classified according to Ericson and Kurol and shows the severity of it by representing which part of the tooth tissues in reference to the pulp are destroyed, rather than numerical values in millimeters, which alone do not represent the clinical severity of ERR.

It is important to notice that multicausation of ERR is more likely than a single causation [26], and therefore, it must be considered that the positional characteristics of an impacted third molar may not be the only risk factors for external root resorption. Inherited tendency for root resorption, physiological characteristics of hard tissue response to pressure, and inflammatory processes may also have a relationship with external root resorption [27].

Staderini et al. in their recent systematic review concluded that there was no sufficient evidence to define absolute indications or contraindications for preventive removal of impacted third molars [28]. Ventä et al. found that the number of disease-free third molars decreased with increasing age, and most dramatically, this occurred among teeth at the cervical level with the neighboring second molar [29]. Only one-fourth of third molars may remain disease-free after the age of 30 years [29]. It is also important not to forget that positional changes and eruption of ITM are an unpredictable phenomena [30]. Bastos et al. found that despite the remarkable agreement for third molar prognosis, oral/maxillofacial surgeons and orthodontists were unable to predict third molars’ eruption by examining a single panoramic radiograph [31]. Therefore, some clinicians consider prophylactic germectomy of the third molar to avoid caries, pericoronitis, or other complications associated with the late extraction of the third molar. Chiapasco et al. found that complications after the removal of mandibular ITM until the age of 24 years were considerably lower in comparison to the complications in older patients [32]. Regarding the postoperative complications following germectomy, only two risk factors are significantly more prevalent in early removal of ITM: female patients are more susceptible to developing postoperative complications, and distally-angled third molars or molars presenting Class III impaction are more prone to cause such complications [28]. An important advantage of germectomy that clinicians should consider is that such a type of intervention is less likely to cause inferior alveolar or lingual nerve damage as the roots of the molar have not yet been fully formed [28]. The results of the present study may be beneficial to dental clinicians by helping to evaluate the particular positional parameters of impacted third molars and make the correct decision regarding extraction or retention of ITM. In cases of ITM mesial inclination, more than 13.6°, or deep impaction in the lower dental arch, clinicians should explain the possible risk of ERR to the patients and propose deeper examination with a CBCT scan for diagnosis of this pathology and treatment decision. Furthermore, in certain cases of young patients, when impaction of the third molar is noticed and certain positional parameters prognose increased risk of ERR in the future, germectomy might be chosen as a method to avoid possible complications.

There are several limitations concerning the findings of this study. The patients included here were strictly filtered according to inclusion criteria and did not represent an average sample of ITM. Moreover, this is a retrospective study where ERR was identified only by CBCT scan, which lacks clinical evaluation. It should be considered that the presence of caries on the distal root surface might complicate the identifications of ERR [12]. Previous studies showed that mesial angulation of ITM increases the risk of caries [33]. However, mostly second molars with no direct tooth-to-tooth contact with ITM were significantly associated with dental caries [9]. In the present study, all ERR were situated at the site of contact between the root surface of the second molar and the third molar crown. Moreover, in a histologic study of eight teeth diagnosed with ERR using conventional intraoral radiography, resorption was confirmed histologically in all the teeth investigated [34]. Results of that study have indicated that the radiographic distinction between ERR and distal caries on second molars in proximity to ITM is generally reliable.

## 5. Conclusions

Based on study results, the following conclusions were drawn:

(1) The incidence of ERR in second molars was significantly associated with the mesial inclination and deep position of ITM.
(2) Depth of ERR did not depend on its location.

## Figures and Tables

**Table 1:** Association of external root resorption of the distal surface of the mandibular second molar with positional characteristics of the impacted third molar.

Variables		ERR on Second Molar Distal Surface					
		Upper Arch			Lower Arch		
		No Resorption *n* = 87	Resorption *n* = 44	*p* Value	No Resorption *n* = 65	Resorption *n* = 58	*p* Value
Mesio-distal position of ITM; *n* (%)	Vertical	39 (70.9%)	16 (29.1%)	<0.001 *	16 (94.1%)	1 (5.9%)	<0.001 *
	Mesioangular	23 (51.1%)	22 (48.49)		44 (49.4%)	45 (50.6%)	
	Horizontal	0 (0%)	4 (100%)		3 (20.0%)	12 (80.0%)	
	Distoangular	25 (92.6%)	2 (7.4%)		2 (100%)	0 (0%)	
Depth of ITM; *n* (%)	Sector A	72 (68.6%)	33 (31.4%)	0.293	55 (59.1%)	38 (40.9%)	0.014 *
	Sector B	15 (57.7%)	11 (42.3%)		10 (33.3%)	20 (66.7%)	
Mesial angulation of ITM; mean (SD)		9.38° (14.31)	22.61° (22.78)	<0.001 *	27.78° (23.02)	49.18° (22.67)	<0.001 *
Available space; *n* (%)	Sufficient	not measured	not measured		4 (57.1%)	3 (42.9%)	0.859
	Reduced	not measured	not measured		36 (54.5%)	29 (44.6%)	
	Insufficient	not measured	not measured		20 (51.3%)	19 (48.7%)	
	No space	not measured	not measured		5 (41.7%)	7 (58.3%)	

* Statistically significant. ERR: external root resorption, ITM: impacted third molars.

**Table 2:** Distribution of the location and severity of external root resorption of the distal surface of second molars.

	Upper Arch (*n* = 44)	Lower Arch (*n* = 58)	*p* Value between Arches
**Location of ERR** (*n* = 102)			0.002 *
Cervical	12 (27.3%)	36 (62.1%)	
Medial	24 (54.5%)	16 (27.6%)	
Apical	8 (18.2%)	6 (10.3%)	
**Severity of ERR** (*n* = 102)			0.236
Slight	29 (65.9%)	47 (81%)	
Moderate	12 (27.3%)	8 (13.8%)	
Severe	3 (6.8%)	3 (5.2%)	

* Statistically significant.

**Table 3:** Association of the severity of external root resorption with the positional characteristics of impacted third molars.

**Severity of ERR (*n* = 102)**	**Depth of Impaction**				***p* Value**
	**Sector A**		**Sector B**		
Slight	61 (85.9%)		15 (48.4%)		<0.001 *
Moderate	7 (9.9%)		13 (41.9%)		
Severe	3 (4.2%)		3 (9.7%)		
**Severity of ERR (*n* = 102)**	**Mesio-Distal Position of ITM**				
	**Vertical**	**Mesioangular**	**Horizontal**	**Distoangular**	
Slight	14 (82.4%)	50 (74.6%)	11 (68.8%)	1 (50.0%)	0.43
Moderate	3 (17.6%)	11 (16.4%)	5 (31.3%)	1 (50.0%)	
Severe	0 (0%)	6 (9.0%)	0 (0%)	0 (0%)	

* Statistically significant.

**Table 4:** Association between location and severity of external root resorption; *p* < 0.001.

Location of ERR (*n* = 102)	Severity of ERR		
	Slight	Moderate	Severe
Cervical	44 (57.9%)	2 (10.0%)	2 (33.3%)
Medial	28 (36.8%)	11 (55.0%)	1 (16.7%)
Apical	4 (5.3%)	7 (35.0%)	3 (50.0%)
